# Population mixing, socioeconomic status and incidence of childhood acute lymphoblastic leukaemia in England and Wales: analysis by census ward

**DOI:** 10.1038/sj.bjc.6604237

**Published:** 2008-02-05

**Authors:** C A Stiller, M E Kroll, P J Boyle, Z Feng

**Affiliations:** 1 Childhood Cancer Research Group, Department of Paediatrics, University of Oxford, Oxford OX2 6HJUK, UK; 2 School of Geography and Geosciences, Irvine Building, University of St Andrews, St Andrews KY16 9AL, UK

**Keywords:** leukaemia, child, population mixing, urbanisation, deprivation, population density

## Abstract

In this population-based study of acute lymphoblastic leukaemia (ALL) diagnosed among children aged under 15 years in England and Wales during 1986–1995, we analysed incidence at census ward level in relation to a range of variables from the 1991 census, which could be relevant to theories of infectious aetiology. ‘Population-mixing’ measures, used as surrogates for quantity and diversity of infections entering the community, were calculated from census data on the origins and destinations of migrants in the year before the census. Incidence at ages 1–4 years tended independently to be higher in rural wards, to increase with the diversity of origin wards from which in-migrants had moved during the year before the census, and to be lower in the most deprived areas as categorised by the Carstairs index. This last association was much weaker when urban/rural status and in-migrants' diversity were allowed for. There was no evidence of association with population mixing or deprivation for ALL diagnosed at ages 0 or 5–14 years. The apparent specificity to the young childhood age group suggests that these associations are particularly marked for precursor B-cell ALL, with the disease more likely to occur when delayed exposure to infection leads to increased immunological stress, as predicted by Greaves. The association with diversity of incomers, especially in rural areas, is also consistent with the higher incidence of leukaemia predicted by Kinlen, where population mixing results in below average herd immunity to an infectious agent.

Acute lymphoblastic leukaemia (ALL) is the most frequent cancer among children in industrialised nations (Parkin *et al*, 1998). Little is known about its aetiology, but there is increasing evidence from epidemiological studies that infection may be important (McNally and Eden, 2004). Incidence is higher among more affluent populations, both internationally and within countries, and the peak of incidence in early childhood is also more marked in more affluent countries (Parkin *et al*, 1998). These patterns are consistent with the hypothesis that precursor B-cell ALL (the most frequent type of childhood ALL, diagnosed mainly between the ages of 1 and 5 years) results from at least two mutations, with the second one being more likely to occur in children in whom delayed exposure to infection led to increased immunological stress (Greaves, 1988).

The hypothesis that some childhood leukaemia could be a rare response to an unidentified infection, with the incidence related to the level of herd immunity in the population at risk, was first tested in a study of the Scottish New Town of Glenrothes (Kinlen, 1988). Subsequently, high incidence rates were found in other UK populations subject to high levels of population mixing, including other rural new towns, areas receiving large numbers of servicemen, migrant construction workers or wartime evacuees, and towns with large increases in the level of commuting (Kinlen, 1995). Other studies have looked for evidence of an effect of less extreme levels of population mixing, measured in various ways (Stiller and Boyle, 1996; Dickinson and Parker, 1999). Kinlen's hypothesis does not specify any subtype of childhood leukaemia. It seems likely, however, that it should apply in particular to precursor B-cell ALL, since that is the most frequent type of childhood leukaemia in the United Kingdom and other western countries.

In the present study we analyse incidence of childhood ALL in England and Wales at 1991 census ward level for the period 1986–1995 in relation to a range of variables which could be relevant to either or both of the two theories of infectious aetiology described above.

## SUBJECTS AND METHODS

### Leukaemia cases

Registrations for ALL diagnosed below the age of 15 years in England and Wales during 1986–1995 were taken from the population-based National Registry of Childhood Tumours (Stiller, 2007). Cases occurring after a previous childhood cancer were excluded. Postcode to census geography look-up tables allowed cases to be assigned to census enumeration districts (the smallest areas for which 1991 census data were released) and wards.

### Population data

Numbers of children in age groups 0, 1–4, 5–9 and 10–14 years in each ward were obtained from the 1991 census, and multiplied by 10 to give estimates of person-years at risk for the 10-year study period (Table 1). While some local authorities produce estimates for intervening years, the methods used to do this vary, and the decennial censuses provide the only consistent and reliable source of data on population counts for small areas.

### Sociodemographic variables

For each ward we calculated the proportions of all residents, of adults aged over 15 years, and of children aged under 15 years, who had been resident outside the ward one year previously, using the Small Area Statistics (SAS) from the 1991 census. The Special Migration Statistics (SMS) of the 1991 census provide separate counts of child and adult migrants between each origin and destination ward within Britain for all people whose ward of residence on census day differed from that one year before. From these data we derived three indices of population mixing, using Shannon's entropy (Shannon, 1948). For each ward the diversity of the wards of origin of incomers into that ward was calculated for incomers of all ages and, separately, for those aged over and under 15 years, using the formula, 
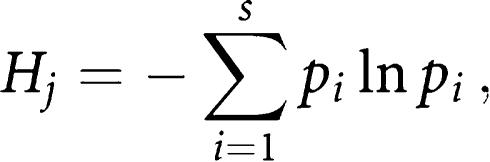
 where *p*_*i*_ is the proportion of all migrants moving into the *j*^th^ area who came from the *i*^th^ area and *s* is the total number of areas. The measure of socioeconomic status was the Carstairs index of deprivation, calculated from variables in the 1991 census SAS at ward level (Carstairs and Morris, 1991). The population density of wards was calculated from the 1991 census statistics. For each of these variables, the wards were classified by quintiles into five groups with approximately equal numbers of wards (Table 2). Wards were also classified as urban (*N*=5525), mixed urban/rural (*N*=2031), or rural (*N*=1953) according to ONS definitions based on land use (Craig, 1987; Office for National Statistics (ONS) and General Register Office for Scotland (GROS), 1997).

### Statistical methods

Possible associations between leukaemia incidence and the variables listed above were investigated using Poisson regression methods. Throughout the analyses a multiplicative model was used.

## RESULTS

There were 3150 new cases of ALL registered during the study period. Table 1 shows national numbers of cases, person-years at risk and incidence rates by age and sex. Incidence was highest in the 1–4 years age group, which accounted for over half of all registrations. There was an excess of boys overall and in every age group except the first year of life, when more girls were affected.

Table 3 shows incidence rate ratios and results of tests for heterogeneity and trend between quintile groups from univariate analyses of incidence in the age groups 0, 1–4 and 5–14 years in relation to percentages of incomers, diversity of incomers, population density and Carstairs deprivation index. There was no evidence that incidence of ALL in any of these age groups, nor at ages 5–9 and 10–14 years (results not shown), was associated either with the percentage of incomers among the total, adult or child population or with population density. There was, however, statistically significant heterogeneity of incidence at ages 1–4 years with the diversity of wards from which in-migrants of all ages combined had originated. There was a suggestion of higher incidence in wards where incomers came from more diverse origins, although the test for trend was nonsignificant. In contrast, incidence was highest in wards where there was least diversity of wards of origin among child incomers, but the test for heterogeneity was marginally nonsignificant and there was no suggestion of a monotonic trend. The strongest heterogeneity effect was for incidence at ages 1–4 years in relation to deprivation. This was also the only variable for which the test for trend was formally significant, but there was little suggestion of a linear trend and the test result was largely due to lower incidence in the most deprived wards. As with the other variables in Table 3, there was no evidence of association between deprivation and incidence at ages other than 1–4 years.

Table 4 shows the results of univariate analyses of incidence in wards classified by degree of urbanisation. Incidence at ages 1–4 years was higher in wards classed as rural than in those classed as urban or mixed; the test for heterogeneity was borderline significant.

Table 5 and 6 show the results of univariate and multivariate analyses of incidence in the age group 1–4 years with various combinations of urbanisation, diversity of total incomers and the Carstairs deprivation index. Urbanisation (model A) and diversity of incomers (model B) had independent effects, and both contributed significantly in a combined model (model D). Adding deprivation to this model (model G) did not significantly improve the fit, although the test for trend was of borderline significance. Deprivation, however, had the most highly significant effect in univariate analysis (model C). Adding urbanisation and diversity of incomers (model G) did not improve the fit significantly.

## DISCUSSION

In this study of childhood ALL throughout England and Wales between 1986 and 1995, incidence among children aged 1–4 years tended to be higher in census wards where in-migrants came from a greater diversity of origins, had a lower deprivation score and were more rural. Among infants and older children, there was little evidence of variation in incidence with any of the sociodemographic factors studied.

The difference in results between age groups and the strong association with affluence are consistent with Greaves's hypothesis. In the United Kingdom, the precursor B-cell subtype accounts for a much higher proportion of ALL in the 1–4 years age group than it does among infants or older children (Stiller, 2007). It therefore seems likely that the observed effects were specific to this subtype, as predicted by Greaves. At least two of the four closely correlated components of the Carstairs deprivation index, namely household overcrowding and lack of use of a car, are likely to be related to early exposure to infection, and the associated reduction in risk is as predicted. When the analyses were repeated for each of these variables separately, the results were very similar to those for the Carstairs index (results not shown).

The independent associations with diversity of incomers and with rural location are consistent with Kinlen's hypothesis, and also with that of Greaves. Both theories predict raised incidence of leukaemia in children who meet new infection relatively late. This requires not only contact with infection in childhood, presumably shortly before the development of leukaemia, but also isolation from infection earlier in life. The strongest effect would be expected in previously isolated areas following a sudden increase in diversity of incomers. Rural areas are more likely to be isolated than urban ones, although there might also be pockets of relative isolation within some urban centres. Our population-mixing model therefore combines diversity of origin of incomers with urbanisation.

It has been suggested that the association of ALL with affluence might be entirely explained by an association between affluence and population mixing, as wealthy communities may tend to be both more mobile and more rural than average. The effect of the Carstairs deprivation index was much weaker when population mixing (defined as the combination of urbanisation and incomers' diversity) was allowed for, but the adjusted risk in the poorest category was still noticeably low, suggesting that at least part of the effect is not explained by population mixing. The effect of the combination of urbanisation and incomers' diversity did not reach statistical significance when the deprivation index was allowed for. Taken together, these results are consistent with the idea that population mixing and the Carstairs index are measures of closely related but not identical processes.

In a study of childhood ALL throughout England and Wales during 1979–1985, incidence at county district level was found to be related to indicators of population mixing and socioeconomic status derived from 1981 census statistics (Stiller and Boyle, 1996). The present study differs in two main ways. First, the geographical units were census wards rather than county districts. Census wards are considerably smaller than county districts, with average child populations of about 1000 and about 25 000, respectively. Furthermore, they are more homogeneous as regards population size: child populations of county districts ranged from 251 to 212 665, with 98% in the range 4499–90 185, whereas those of census wards range from 0 to 9739, with 98% in the range 127–3737. The degree of heterogeneity of socioeconomic and migration-related variables in small areas within wards should be considerably less than that within districts. Also, particularly in urban areas, much migration between wards does not involve a move between districts (Boyle *et al*, 1998; Parslow *et al*, 2002); this sizeable component of migration would have been ignored in our earlier study. Second, the use of sociodemographic and migration data from the 1991 census made it possible to examine separately the effects of diversity of origin of incomers within the adult and childhood age groups, which was not possible in our previous study.

Significant positive trends in incidence of ALL at ages 0–4 and 5–9 years were previously found with the proportion of recent incomers among the child population of a district (Stiller and Boyle, 1996). The combination of higher migration of people of all ages and greater diversity of their districts of origin was also associated with higher incidence in both age groups. In the present study, there was no evidence that incidence of childhood ALL was related to volume of migration, but there was again a positive association with diversity of incomers of all ages and incidence of ALL diagnosed at ages 1–4 years. A similar pattern was found with diversity of origin of adult incomers, although the association was not significant. This is consistent with the results of several of Kinlen's studies in which the relevant population mixing was necessarily attributable to adults, because it was defined in terms of employment (Kinlen *et al*, 1995; Kinlen, 1997) or military service (Kinlen and Hudson, 1991). Similar results were, however, found in Kinlen's studies of rural new towns (Kinlen *et al*, 1990) and of rural areas receiving large numbers of evacuees in wartime (Kinlen and John, 1994), where the population mixing was attributable substantially or even totally to children. In the present study, by contrast, wards with least diversity of areas of origin among child migrants had a higher incidence of ALL. The reasons for this are unclear, but it seems likely that, during the present study period, very few wards experienced child population mixing of such extreme intensity as that encountered by the populations in some of Kinlen's studies. One possible explanation is that the effect of increased population mixing involving adults is enhanced in areas with the lowest levels of child population mixing, where the child population may tend to have impaired herd immunity through low diversity of exposure to infections usually transmitted by children.

The incidence of leukaemia and other childhood cancers in Yorkshire during 1986–1996 was examined at ward level in relation to population mixing (Parslow *et al*, 2002). The study period was very similar to that of the present study but the study region contained less than one-tenth of the total population of England and Wales. As in the present study, proportions of incomers to each ward and the diversity of their wards of origin were derived from census data. The final model also included deprivation, as measured by the Townsend index, and population density. Results were presented for ALL at all ages under 15 years combined. An inverse relation was found between diversity of incomers and incidence of ALL, both for children and for incomers of all ages, whereas nationally, there was a tendency for incidence to increase with diversity of total and adult incomers. This suggests that either the effects of population mixing at all ages combined, and particularly among adults, are not uniform across the whole country, or that the result in Yorkshire was a chance finding, perhaps attributable to the relatively small number of cases studied.

The UK Childhood Cancer Study (UKCCS) also found a significantly raised risk of ALL with low diversity of origins of migrants in England, Scotland and Wales during 1991–1996 (Law *et al*, 2003). Although the UKCCS would have had many cases in common with the present study, direct comparison between the two sets of results is difficult for several reasons. Unlike the present study, and others reviewed here, the UKCCS had a case–control design, the controls being matched with cases on National Health Service organisational units of residence, whose mean child population was 100 000. This would undoubtedly have introduced an element of overmatching on socioeconomic status, population density and migration pattern and, while not necessarily a source of bias, would tend to impair the ability of the analysis to detect effects of these variables. The raised odds ratio for low diversity of total incomers in univariate analysis was almost unchanged in a multivariate model that also included an index of deprivation at the level of census enumeration district, suggesting that the effects of the two variables were independent. Deprivation was very nearly statistically significant as a risk factor for ALL, with a tendency for risk to be higher in more affluent areas.

Several studies have examined related variables in other populations or time periods. In the United States, Adelman *et al* (2007) found a significantly raised risk of ALL at ages 0–4 years in counties where at least half of the residents had changed address during a 5-year period. In Ontario province (Canada), Koushik *et al* (2001) employed percentage population change as a measure of mixing, with higher levels of change arising predominantly from migration. A higher incidence of leukaemia, particularly ALL, was found in rural areas with more marked population growth but there was no evidence of raised incidence in urban areas with similarly high levels of population increase. This is perhaps analogous to the present study, in which incidence was highest in rural areas with high diversity of incomers; migrants' origins may well be more diverse in Canada than in the United Kingdom.

While many previous studies have found a population-mixing effect in rural areas, often among affluent populations, the effects need not be restricted to these areas. In England and Wales, Dickinson *et al* (2002) studied incidence of leukaemia and non-Hodgkin lymphoma (NHL) during 1966–1987 using migration data from the 1981 census. They found a higher incidence in wards with higher proportions of incomers, although this was largely restricted to urban areas. Satisfactory migration data were not available from the 1971 census, however, and the question of how consistent migration patterns were throughout the study period remains unanswered. Moreover, ‘leukaemia and NHL’ is a very disparate group. A weak association of childhood leukaemia incidence with proximity to railways in the same data set was tentatively attributed to population mixing in two deprived wards with high proportions of incomers in their populations (Dickinson *et al*, 2003).

Numerous studies have shown an elevated risk of childhood leukaemia or ALL in areas of higher socioeconomic status (Githens *et al*, 1965; Alexander *et al*, 1990a; Draper *et al*, 1991; Stiller and Boyle, 1996; Borugian *et al*, 2005). In our 1979–1985 study, it was suggested that the socioeconomic gradient might be largely due to population mixing (Stiller and Boyle, 1996), but the present study showed a substantial effect of socioeconomic status after urbanisation and incomers' diversity had been allowed for (see above).

No significant variation in incidence by population density was found in the present study. There was a borderline significant variation by urban/rural status at ages 1–4 years, with incidence tending to be higher in rural areas, and the difference became significant after allowing for diversity of total incomers. It should be emphasised that the classification of urbanisation was based on land use rather than population density, although obviously urban areas tended to have higher population density than rural ones. Previous studies in Britain have also found a higher incidence of ALL in rural or isolated areas (Alexander *et al*, 1990b; Dickinson and Parker, 1999). This contrasts with findings of higher incidence in urban areas in several other countries, including Greece (Petridou *et al*, 1997), Taiwan (Li *et al*, 1998), Sweden (Hjalmars *et al*, 1999) and the United States (Adelman *et al*, 2005). None of these studies controlled for socioeconomic status, however, and patterns of socioeconomic status in relation to urbanisation may differ between countries.

In conclusion, the results of the present study are consistent with the hypotheses of both Kinlen and Greaves. The apparent specificity of the association to the young childhood age group suggests that the effect is particularly marked for the precursor B-cell subtype, as predicted by Greaves. The association with incomers' diversity, particularly in rural areas, is as predicted by Kinlen. Both this and the strong association with the deprivation index are also consistent with Greaves's hypothesis.

This study provides further evidence that the risk of precursor B-cell ALL in children may be increased by delayed exposure to unknown common infection(s), following relative geographic or social isolation early in life.

## Figures and Tables

**Table 1:** ALL among the child population of England and Wales 1986–1995

	**Age group (years)**				
	**0**	**1–4**	**5–9**	**10–14**	**0–14**
*Males*					
No. of cases	49	937	490	298	1774
Person-years (10^6^)	3.45	13.51	16.00	15.32	48.28
Incidence	14.2	69.3	30.6	19.4	38.1
					
*Females*					
No. of cases	73	729	375	199	1376
Person-years (10^6^)	3.30	12.91	15.23	14.56	45.99
Incidence	22.1	56.5	24.6	13.7	31.1
					
*Total*					
No. of cases	122	1666	865	497	3150
Person-years (10^6^)	6.75	26.42	31.23	29.88	94.27
Incidence	18.1	63.1	27.7	16.6	34.7

ALL=acute lymphoblastic leukaemia.

Numbers of cases, person-years at risk and annual incidence rates per million children. Rates for ages 0–14 years are age standardised according to the world standard population.

**Table 2:** Classification of continuous variables

	**Minimum**	**Maximum in each quintile category**				
**Variable**	**—**	**1**	**2**	**3**	**4**	**5**
% incomers in total population	1.45	4.95	5.92	6.89	8.33	29.45
% incomers in adult population	0.00	4.96	5.96	6.98	8.54	33.76
% incomers in child population	0.61	4.40	5.48	6.54	8.04	26.67
Diversity of total incomers	0.96	3.38	3.65	3.92	4.26	6.33
Diversity of adult incomers	1.05	3.44	3.71	3.98	4.32	6.14
Diversity of child incomers	0.00	2.25	2.62	2.91	3.20	4.64
Population density (persons per km^2^)	2	87	463	1632	3531	20 311
Carstairs deprivation score	−5.33	−2.80	−1.72	−0.28	2.12	24.58

**Table 3:** Incidence rate ratios (IRRs) from univariate models for ALL by age group for wards classified according to quintiles of the following variables: percentage of incomers in total, adult and child population; diversity of total, adult and child incomers; population density; and Carstairs deprivation score

**Age group (years)**	**Significance level**		**IRR in each quintile category**				
**Variable**	**Heterogeneity**	**Trend**	**1**	**2**	**3**	**4**	**5**
*Age 0*							
% incomers in total population	0.89	0.95	1	1.09	1.17	1.21	0.93
% incomers in adult population	0.36	0.94	1	0.84	1.42	1.09	0.89
% incomers in child population	0.24	0.17	1	1.22	1.14	0.64	0.83
Diversity of total incomers	0.45	0.80	1	1.33	1.42	0.87	1.20
Diversity of adult incomers	0.97	0.90	1	1.05	1.20	1.00	1.02
Diversity of child incomers	0.31	0.13	1	0.75	0.87	0.89	0.57
Population density	0.53	0.36	1	2.26	2.40	2.06	2.40
Carstairs deprivation score	0.85	0.87	1	0.78	1.13	1.05	0.96
							
*Ages 1–4*							
% incomers in total population	0.96	0.75	1	1.00	0.99	0.95	1.00
% incomers in adult population	0.70	0.82	1	1.07	0.99	0.96	1.03
% incomers in child population	0.95	0.97	1	1.03	0.98	1.04	1.00
Diversity of total incomers	0.04	0.11	1	1.14	1.01	1.24	1.13
Diversity of adult incomers	0.13	0.18	1	1.07	1.00	1.20	1.08
Diversity of child incomers	0.08	0.85	1	0.78	0.76	0.84	0.86
Population density	0.29	0.26	1	0.93	0.81	0.84	0.86
Carstairs deprivation score	0.011	0.0014	1	1.01	0.99	0.94	0.82
							
*Ages 5–14*							
% incomers in total population	0.64	0.34	1	1.01	1.11	1.02	1.09
% incomers in adult population	0.53	0.40	1	0.95	1.06	0.97	1.07
% incomers in child population	0.97	0.91	1	1.04	1.01	0.99	1.02
Diversity of total incomers	0.97	0.89	1	0.94	0.95	0.94	0.97
Diversity of adult incomers	0.99	0.96	1	0.98	1.00	0.97	1.01
Diversity of child incomers	1.00	0.83	1	0.97	0.97	0.95	0.97
Population density	0.87	0.99	1	1.06	1.12	1.08	1.05
Carstairs deprivation score	0.97	0.73	1	0.96	1.00	0.95	0.97

ALL=acute lymphoblastic leukaemia.

For each variable, the reference group is the lowest quintile category (1).

Significance levels from tests for heterogeneity and trend in relative risk between categories are also provided.

**Table 4:** Incidence rate ratios (IRRs) for ALL by age group for wards classified by urban/rural status

	**Significance level**	**IRR for wards grouped by urban/rural status**		
**Age group (years)**	**Heterogeneity**	**Urban**	**Mixed**	**Rural**
0	0.40	1	1.20	0.63
1–4	0.0507	1	1.07	1.26
5–14	0.89	1	0.97	1.03
Number of wards		5525	2031	1953

ALL=acute lymphoblastic leukaemia.

The reference group is the urban category.

Significance levels from tests for heterogeneity of relative risk between categories are also provided.

**Table 5:** Incidence rate ratios (IRRs) from univariate and multivariate models for ALL diagnosed at ages 1–4 years, for wards classified by urban/rural status and by quintiles of the following variables: diversity of total incomers and Carstairs deprivation score

	**Significance level**	**Urban/rural status**			**Diversity of total incomers**					**Carstairs deprivation score**				
**Model**	**Heterogeneity**	**Urban**	**Mixed**	**Rural**	**1**	**2**	**3**	**4**	**5**	**1**	**2**	**3**	**4**	**5**
A	0.051	1	1.07	1.26	—	—	—	—	—	—	—	—	—	—
B	0.042	—	—	—	1	1.14	1.01	1.24	1.13	—	—	—	—	—
C	0.011	—	—	—	—	—	—	—	—	1	1.01	0.99	0.94	0.82
D	0.008	1	1.07	1.31	1	1.15	1.02	1.27	1.17	—	—	—	—	—
E	0.018	1	1.02	1.17	—	—	—	—	—	1	1.01	1.00	0.97	0.84
F	0.008	—	—	—	1	1.12	0.97	1.18	1.08	1	1.01	0.99	0.95	0.83
G	0.007	1	1.03	1.22	1	1.13	0.99	1.21	1.12	1	1.02	1.01	0.99	0.87

ALL=acute lymphoblastic leukaemia.

The reference groups are the urban category and the lowest quintile categories (1), respectively.

Significance levels from tests for heterogeneity of relative risk between categories are also provided.

**Table 6:** Comparison of models defined in Table 5

	**Comparison**		**Significance level**	
**Adjusted model**	**Model 1**	**Model 2**	**Heterogeneity**	**Trend**
Diversity of total incomers, adjusting for urban/rural status	A	D	0.022	0.042
Urban/rural status, adjusting for diversity of total incomers	B	D	0.023	—
Urban/rural status and diversity of incomers, adjusting for Carstairs	C	G	0.087	—
Carstairs, adjusting for urban/rural status and diversity of incomers	D	G	0.154	0.052

Significance levels from comparisons of model 1 with model 2 (heterogeneity), or model 1 with a variant of model 2 that treat the levels of the extra factor as a continuous variable (trend).
